# Infectious disease hospitalization after receipt of human papillomavirus vaccine: a nationwide register-based cohort study among Danish, Finnish, Norwegian, and Swedish girls

**DOI:** 10.1007/s10654-024-01197-3

**Published:** 2025-01-06

**Authors:** Ida Laake, Berit Feiring, Lise Gehrt, Hélène Englund, Mika Lahdenkari, Signe Sørup, Heta Nieminen, Lill Trogstad

**Affiliations:** 1 Division of Infection Control, Norwegian Institute of Public Health, Oslo, Norway; 2 Bandim Health Project, Research Unit Open, Department of Clinical Research, University of Southern Denmark, Odense C, Denmark; 3 Danish Institute for Advanced Study, University of Southern Denmark, Odense C, Denmark; 4 Department of Public Health Analysis and Data Management, Public Health Agency of Sweden, Solna, Sweden; 5 Knowledge Department, Finnish Institute for Health and Welfare, Helsinki, Finland; 6 Department of Clinical Epidemiology, Department of Clinical Medicine, Aarhus University Hospital and Aarhus University, Aarhus, Denmark; 7 Norwegian Institute of Public Health, PO Box 222, Skøyen, Oslo, 0213 Norway

**Keywords:** HPV vaccine, Non-specific vaccine effects, Registry-based cohort, Nordic countries

## Abstract

It has been suggested that non-live vaccines may increase susceptibility to non-targeted infections and that such deleterious non-specific effects are more pronounced in girls. We investigated whether receipt of non-live vaccine against human papillomavirus (HPV) was associated with increased risk of infectious disease hospitalization. A nationwide cohort study based on detailed individual-level data from national registries was performed in Denmark, Finland, Norway, and Sweden. The cohort consisted of girls aged 11–14 years in Denmark, Finland, and Norway, and 10–14 years in Sweden. Cox regression, with extensive control for potential confounders, was used to assess whether risk of infectious disease hospitalization with at least one overnight stay differed according to time-varying HPV vaccination status. In total, 754 458 girls were included in the analysis. The infectious disease hospitalization rate (per 10 000 person years) was 44.1 in Denmark, 35.7 in Finland, 37.1 in Norway, and 28.5 in Sweden. Comparing HPV-vaccinated with HPV-unvaccinated person time, the adjusted hazard ratio (95% confidence interval) was 0.81 (0.72, 0.90) in Denmark, 0.69 (0.60, 0.80) in Finland, 0.76 (0.66, 0.88) in Norway, and 0.59 (0.49, 0.71) in Sweden. Decreased risk was observed regardless of number of doses, except in Norway, where risk among girls with only one dose did not differ from risk among unvaccinated girls. Receipt of HPV vaccine was consistently associated with decreased risk of infectious disease hospitalization among girls in the Nordic countries. Our study does not support that HPV vaccines have deleterious non-specific effects.

**Supplementary Information:**

The online version contains supplementary material available at 10.1007/s10654-024-01197-3.

## Introduction

Vaccines provide effective protection against many infectious diseases. In addition to the specific effects on the targeted diseases, vaccines may also have non-specific effects on mortality and morbidity caused by infections not targeted by the vaccine [1, 2]. Live vaccines, such as measles vaccine, may provide protection against non-targeted infections. In contrast, it has been suggested that non-live vaccines such as vaccine against diphtheria, tetanus, and pertussis (DTP) may increase susceptibility to non-targeted infections and that these effects are more pronounced in girls [2, 3]. Moreover, co-administration of live and non-live vaccines has been suggested to attenuate the potential deleterious non-specific effects of the non-live vaccine [2].

There is limited evidence on non-specific effects of non-live vaccines other than DTP vaccine. Non-live vaccines against human papillomavirus (HPV) are offered to girls in early adolescence to protect against cervical cancer and precancerous lesions [4–7]. Only two previous studies have examined non-targeted infections after HPV vaccination but did not find any indication of increased risk [8, 9]. The extensive data on the safety of HPV vaccines are reassuring, and the Global Advisory Committee on Vaccine Safety has not identified any safety concerns [10]. Nevertheless, unsupported claims that HPV vaccine can cause certain chronic conditions like complex regional pain syndrome and postural orthostatic tachycardia syndrome have led to an HPV vaccination crisis in several countries [11–13]. Thorough evaluation of potential harmful effects of HPV vaccine is therefore crucial to maintain public confidence in HPV vaccination programs.

The aim of this study was to investigate whether receipt of an HPV vaccine is associated with increased risk of infectious disease hospitalization in adolescent girls in four Nordic countries.

## Materials and methods

### Design and data sources

Using detailed individual-level data from national registries and databases, we performed a nation-wide cohort study in Denmark, Finland, Norway, and Sweden. In these countries, girls are offered free-of-charge HPV vaccination through the national childhood immunization programs (NCIPs). The vaccine is offered at age 12 years in Denmark, Finland, and Norway, and at age 11 or 12 years in Sweden. Throughout the study period (defined below), Finnish girls were offered the bivalent vaccine Cervarix^®^, targeting HPV16 and HPV 18, while Swedish girls were offered the quadrivalent vaccine Gardasil^®^, targeting HPV6, HPV11, HPV16, and HPV 18. In Denmark and Norway, girls were initially offered Gardasil^®^, but towards the end of the study period, the vaccine used in the programs changed to Cervarix^®^. Further details of the HPV vaccination programs are presented in Table 1. Furthermore, vaccines offered through the NCIPs between ages 4 and 15 years to girls in the birth cohorts eligible for HPV vaccination are presented in Supplementary Tables 1–4. Information on vaccinations was obtained from The Danish Vaccination Register, the Finnish vaccination register, the Norwegian Immunization Registry, and the National Vaccination Register in Sweden [21–24]. The registries contain information on vaccines in the NCIP since 1996 in Denmark, 2009 in Finland, 1995 in Norway, and 2013 in Sweden.

**Table 1:** HPV vaccine in the national childhood immunization programs in the Nordic countries during the study period

	Introduction	Delivery strategy	Primary target group	Vaccine offered	Recommended schedule	Note
Denmark	January 2009 [14]	General practitioners	12-year-old girls	Until February 1, 2016: Gardasil^®^ From February 1, 2016: Cervarix^®^ [15]	Until February 1, 2016: Three doses at 0, 2, 6 months. From February 1, 2016: Two doses with a minimum interval of 5 months.	Until 2016, revaccination with live MMR vaccine was also recommended at age 12 years. HPV vaccine could be given together with other vaccines [14].
Finland	November 2013 [16]	School-based	Girls in 6th grade (approximately 12 years old)^a^	Cervarix^®^	Until August, 2016: Three doses at 0, 1, 6 months. From August, 2016^b^: Two doses with a minimum interval of 5 months [17].	
Norway	August 2009^c^ [18]	School-based	Girls in 7th grade (approximately 12 years old). From August 2018^d^: All children in 7th grade.	Until August, 2017: Gardasil^®^ From August, 2017^e^: Cervarix^®^ [19]	Until August, 2017: Three doses at 0, 2, 6 months. From August, 2017^e^: Two doses with a minimum interval of 6 months.	
Sweden	January 2012 [20]	School-based	Girls in 5th or 6th grade (approximately 11 or 12 years old, respectively)	Gardasil^®^	Until July 2015: Three doses at 0, 2, 6 months. From July 2015: Two doses with a minimum interval of 5 months [20].	

HPV: human papillomavirus; MMR: measles, mumps, rubella

^a^ During the first two years of the program, HPV vaccine was also offered to girls in grades 7 to 9 (aged 13 − 15 years) in a catch-up program

^b^ From school year 2016/17. The school year in Finland starts around August 10

^c^ HPV vaccine first offered during school year 2009/2010. The school year in Norway starts around August 20

^d^ HPV vaccine offered to all children in 7th grade from school year 2018/19

^e^ Two doses of Cervarix^®^ offered from school year 2016/17

Information on hospital contacts was obtained from patient registries with national coverage and individual-level data since 1978 in Denmark, 1994 in Finland, 2008 in Norway, and 1987 in Sweden [25]. Information on redeemed prescriptions, demographics, socioeconomic factors, and residency was also obtained. Data from the different registers was linked using the unique personal identifier assigned to all residents in the Nordic countries.

A description of the common data model used to generate homogenous country-specific data sets has been published previously [26]. Due to data protection legislation, the data was stored and analyzed separately in each country.

### Study period

In Denmark and Finland, the study period started when HPV vaccine was introduced into the NCIP; January 1, 2009, and November 1, 2013, respectively. In Norway, the study period started January 1, 2010, the first date with complete information on all covariates (described below). In Sweden, it started January 1, 2013, when the National Vaccination Register was launched. The end of the study period was defined as the last date with available information from all the registries: December 31, 2017, in Denmark, Finland, and Sweden; and December 31, 2018, in Norway.

### Study population and follow-up

We included girls from the birth cohorts offered HPV vaccine through the NCIPS during the study period: 1996–2004 in Denmark; 1998–2004 in Finland; 1999–2005 in Norway; 2003–2004 in Sweden. The study was limited to Danish, Finnish, and Norwegian girls aged 11–14 years and Swedish girls aged 10–14 years. Baseline was defined as the date the girls turned 11 years in Denmark, Finland, Norway, and the date they turned 10 years in Sweden, i.e., approximately 1 year prior to when HPV vaccine was usually offered. To be eligible for inclusion, we required that girls were registered as residents at baseline, to ensure that potential HPV vaccination could be captured. Moreover, Finnish girls born abroad were not eligible, since migration history before 2014 was incomplete and residency at baseline could therefore not be verified [26].

Follow-up started at baseline or at start of the study period, whichever occurred last. Girls who died or emigrated between baseline and start of the study period were excluded from the study. Follow-up ended at emigration, death, age 15 years, or end of the study period, whichever occurred first.

### Outcome

The main outcome was infectious disease hospitalization, defined as an inpatient hospital contact with at least one overnight stay and a primary or secondary diagnosis classified as an infection (Supplementary Table 5). Furthermore, we defined a secondary outcome, hospital contacts with at least one overnight stay and a diagnosis classified as a respiratory tract infection (RTI). Infectious disease hospitalization was treated as a recurrent event. Thus, we included all hospitalizations occurring at least 14 days after discharge of a previous infectious disease hospitalization. Time from admission up to the 14th day after discharge was excluded from analyses, since outcomes cannot occur in this time period [27].

### HPV vaccination

Girls were considered vaccinated from receipt of the first dose of HPV vaccine until the end of follow-up. Girls who received HPV vaccine before start of follow-up, or received a fourth dose of HPV vaccine during follow-up were excluded from the study.

### Covariates

#### Time-independent covariates

Using information from the year the girls turned 10 years of age, we defined household income level, number of children in the household, having a single parent, and maternal educational level. Because information from the registries was not complete, these variables included an additional missing value category. Due to very small missing value categories (< 0.5%), we excluded Finnish girls with missing information on maternal education and Norwegian girls with missing information on household income. In addition, we defined a variable for immigrant background. Furthermore, we defined variables for previous infectious disease hospitalization and for number of previous antibiotic treatment episodes, using information on hospitalizations and prescriptions from a 2-year period prior to baseline (from age 9 to 11 years in Finland, Denmark, and Norway; from age 8 to 10 years in Sweden). Girls who immigrated during the pre-baseline period were excluded due to incomplete information on these covariates.

#### Time-dependent covariates

We defined variables for receipt of diphtheria, tetanus, acellular pertussis (DTaP) booster vaccine (Finland, Sweden), diphtheria, tetanus, acellular pertussis, inactivated polio (DTaP-IPV) booster vaccine (Norway), and MMR vaccine (Denmark and Norway), i.e., vaccines in the NCIPs that the girls in the study population could have been offered during the study period. We also defined receipt of influenza vaccine (Finland), and receipt of non-program vaccines (Denmark, Finland, Norway, data not available in Sweden). Finally, we used all hospital contacts from the start of the pre-baseline period until the end of follow-up to define presence of chronic disease, including congenital and acquired conditions such as congenital heart disease, chromosomal abnormalities, cancer, autoimmune disease, and epilepsy. The complete list of diagnoses can be found in the study by Kristensen et al. [28].

Detailed definitions of the covariates are provided in Supplementary Table 6. The variable assessment periods are illustrated in Supplementary Figs. 1 and 2.

### Statistical analysis

To evaluate the association between HPV vaccination and risk of infectious disease hospitalization we estimated hazard ratios (HRs) and 95% confidence intervals (CIs) with Cox regression. We used stratified Cox models with calendar year of birth as strata and age in days as the underlying time scale. To account for recurrent events, we used the Andersen-Gill generalization of the Cox model, applying a robust variance estimate [29]. Multivariable models were adjusted for the covariates described above. We also included time-dependent variables for season, defined as spring (March–May), summer (June–August), autumn (September–November), and winter (December–February). We performed a sensitivity analysis limited to girls without immigrant background. Since the vaccine used in the program in Denmark and Norway changed during the study period, we also performed a sensitivity analysis limited to birth cohorts offered Gardasil^®^ (1996–2003 in Denmark, 1999–2004 in Norway). In this analysis, girls were censored at receipt of the first dose of HPV vaccine if this occurred after the change of vaccine was implemented.

Furthermore, we evaluated the effect of number of received doses. In Denmark, Finland, and Norway, this analysis was restricted to girls from birth cohorts eligible for the three-dose schedule (1996–2003 in Denmark, 1998–2003 in Finland, 1999–2004 in Norway). Girls were censored at receipt of the first dose of HPV vaccine if this occurred after the two-dose schedule was implemented. In Sweden, the entire study population was eligible for the two-dose schedule, and girls were therefore censored at receipt of the third dose. The follow-up time for each dose was further categorized according to time since vaccination (1–90 days, 91–180 days, 181–365 days, and > 365 days).

As many Danish girls received HPV and MMR vaccines together, we performed an additional analysis in Denmark to assess whether co-administration was associated with lower risk of infectious disease hospitalization compared to receiving the first dose of HPV vaccine after MMR vaccine. This analysis was limited to girls who had received MMR vaccine at age 11 years or later and the first dose of HPV vaccine, either after or together with MMR vaccine. Start of follow-up was at receipt of first dose of HPV vaccine. We included a time-dependent variable indicating receipt of additional HPV vaccine doses, in addition to the same covariates as in the main analysis.

The analyses were performed using Stata/SE 16.0 and 17.0 (StataCorp, College Station, Texas, USA).

## Results

**Fig. 1:**
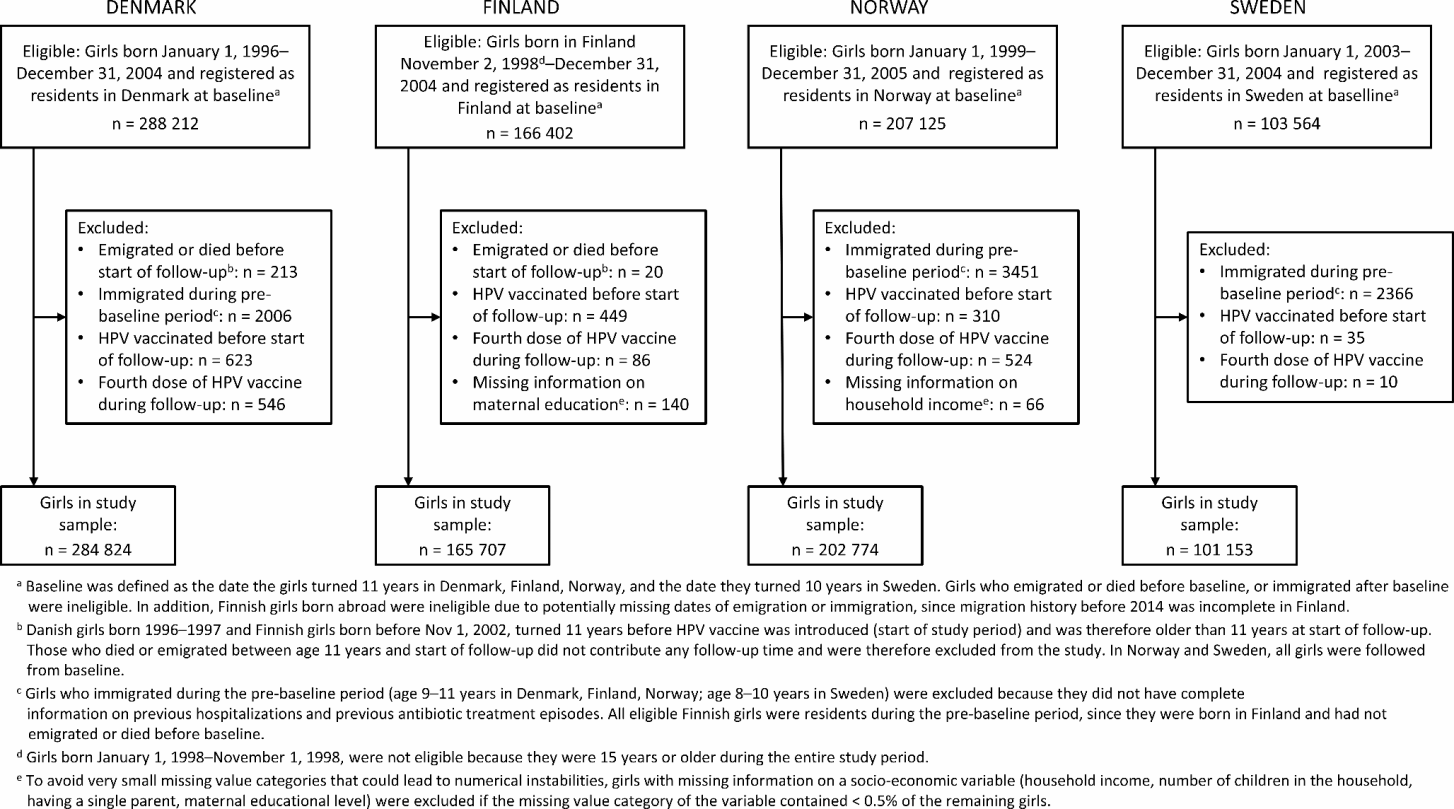
Flow chart of Danish, Finnish, Norwegian, and Swedish girls

A total of 765 303 girls were eligible for inclusion in the study (Fig. 1). We excluded 10 845 girls. The remaining 754 458 girls were included in the analyses: 284 824 in Denmark, 165 707 in Finland, 202 774 in Norway, and 101 153 in Sweden.

Mean follow-up time was 3.5 years in Denmark, 2.4 years in Finland, 3.7 years in Norway, and 4.0 years in Sweden. The proportion of girls who had received at least one dose of HPV vaccine by the end of follow-up was 85.1% in Denmark, 67.3% in Finland, 88.2% in Norway, and 80.5% in Sweden (Table 2). In all countries, girls who did not receive HPV vaccine were more likely than HPV vaccinated girls to have immigrant background, belong to households with low income and more than 4 children, have a mother with low education, and to have a chronic disease.

**Table 2:** Characteristics of girls from Denmark, Finland, Norway, and Sweden included in respective study samples according to HPV vaccination status by end of follow-up

	Denmark		Finland		Norway		Sweden	
	Vaccinated^a^	Unvaccinated^b^	Vaccinated^a^	Unvaccinated^b^	Vaccinated^a^	Unvaccinated^b^	Vaccinated^a^	Unvaccinated^b^
**N (%)**	242 319 (85.1)	42 505 (14.9)	111 463 (67.3)	54 244 (32.7)	178 932 (88.2)	23 842 (11.8)	81 465 (80.5)	19 688 (19.5)
**Age at vaccination**,** mean (SD)**	12.4 (0.6)	–	13.0 (0.9)	–	12.3 (0.3)	–	11.6 (0.6)	–
**Year of birth**,** n (%)**								
1996	29 495 (12.2)	2478 (5.8)	–	–	–	–	–	–
1997	30 187 (12.5)	2180 (5.1)	–	–	–	–	–	–
1998	29 828 (12.3)	1890 (4.4)	1597 (1.4)	2390 (4.4)	–	–	–	–
1999	30 029 (12.4)	1828 (4.3)	15 313 (13.7)	11 947 (22.0)	24 280 (13.6)	5050 (21.2)	–	–
2000	30 273 (12.5)	1892 (4.5)	17 967 (16.1)	8807 (16.2)	24 801 (13.9)	4565 (19.1)	–	–
2001	27 855 (11.5)	3639 (8.6)	17 966 (16.1)	8775 (16.2)	24 793 (13.9)	3711 (15.6)	–	–
2002	25 562 (10.5)	5164 (12.1)	19 309 (17.3)	7054 (13.0)	25 258 (14.1)	2934 (12.3)	–	–
2003	20 803 (8.6)	10 347 (24.3)	19 227 (17.2)	7909 (14.6)	25 992 (14.5)	2895 (12.1)	41 281 (50.7)	8857 (45.0)
2004	18 287 (7.5)	13 087 (30.8)	20 084 (18.0)	7362 (13.6)	26 780 (15.0)	2414 (10.1)	40 184 (49.3)	10 831 (55.0)
2005	–	–	–	–	27 028 (15.1)	2273 (9.5)	–	–
**Immigrant background**,** n (%)**								
Yes	22 364 (9.2)	5047 (11.9)	2388 (2.1)	2163 (4.0)	20 038 (11.2)	4048 (17.0)	13 229 (16.2)	4331 (22.0)
**Household income quintile**^**c**^, **n (%)**								
1 (lowest)	42 210 (17.4)	10 560 (24.8)	20 115 (18.0)	12 631 (23.3)	31 878 (17.8)	6586 (27.6)	13 113 (16.1)	4314 (21.9)
2	45 206 (18.7)	8480 (20.0)	20 580 (18.5)	12 260 (22.6)	35 467 (19.8)	5152 (21.6)	15 315 (18.8)	4445 (22.6)
3	46 693 (19.3)	7326 (17.2)	21 930 (19.7)	11 126 (20.5)	36 900 (20.6)	4247 (17.8)	16 850 (20.7)	4034 (20.5)
4	47 120 (19.4)	6662 (15.7)	23 260 (20.9)	9528 (17.6)	37 515 (21.0)	3949 (16.6)	17 350 (21.3)	3569 (18.1)
5 (highest)	46 456 (19.2)	6381 (15.0)	24 619 (22.1)	8081 (14.9)	37 172 (20.8)	3908 (16.4)	17 702 (21.7)	2939 (14.9)
Missing	14 634 (6.0)	3096 (7.3)	959 (0.9)	618 (1.1)	–	–	1135 (1.4)	387 (2.0)
**Number of children in the household**^**c**^, **n (%)**								
1	30 376 (12.5)	6571 (15.5)	15 964 (14.3)	7451 (13.7)	29 883 (16.7)	4390 (18.4)	14 293 (17.5)	3959 (20.1)
2	125 989 (52.0)	20 681 (48.7)	49 723 (44.6)	20 318 (37.5)	85 588 (47.8)	10 115 (42.4)	40 380 (49.6)	9020 (45.8)
3	65 647 (27.1)	10 707 (25.2)	30 412 (27.3)	13 631 (25.1)	49 873 (27.9)	6483 (27.2)	19 576 (24.0)	4340 (22.0)
≥ 4	20 307 (8.4)	4546 (10.7)	15 364 (13.8)	12 844 (23.7)	13 588 (7.6)	2854 (12.0)	7216 (8.9)	2369 (12.0)
**Single-parent household**^**c**^, **n (%)**								
No	195 647 (80.7)	32 004 (75.3)	90 533 (81.2)	42 753 (78.8)	146 033 (81.6)	18 851 (79.1)	63 318 (77.7)	14 516 (73.7)
Yes	44 695 (18.4)	9907 (23.3)	19 663 (17.6)	10 700 (19.7)	31 304 (17.5)	4779 (20.0)	17 012 (20.9)	4785 (24.3)
Missing	1977 (0.8)	594 (1.4)	1267 (1.1)	791 (1.5)	1595 (0.9)	212 (0.9)	1135 (1.4)	387 (2.0)
**Maternal education**^**c**^, **n (%)**								
Low	38 724 (16.0)	8265 (19.4)	9676 (8.7)	7538 (13.9)	30 106 (16.8)	4451 (18.7)	10 164 (12.5)	2941 (14.9)
Medium	105 428 (43.5)	17 114 (40.3)	41 730 (37.4)	25 408 (46.8)	61 997 (34.6)	8207 (34.4)	28 963 (35.6)	7353 (37.3)
High	94 515 (39.0)	16 098 (37.9)	60 057 (53.9)	21 298 (39.3)	84 295 (47.1)	10 491 (44.0)	31 339 (38.5)	6117 (31.1)
Missing	3652 (1.5)	1028 (2.4)	–	–	2534 (1.4)	693 (2.9)	10 999 (13.5)	3277 (16.6)
**Previous infectious disease hospitalization**^**d**^, **n (%)**								
0	239 972 (99.0)	41 977 (98.8)	110 669 (99.3)	53 721 (99.0)	177 644 (99.3)	23 586 (98.9)	80 907 (99.3)	19 538 (99.2)
≥ 1	2347 (1.0)	528 (1.2)	794 (0.7)	523 (1.0)	1288 (0.7)	256 (1.1)	558 (0.7)	150 (0.8)
**Previous antibiotic treatment episodes**^**e**^, **n (%)**								
0	165 812 (68.4)	29 938 (70.4)	65 837 (59.1)	32 964 (60.8)	141 304 (79.0)	19 068 (80.0)	57 555 (70.6)	13 912 (70.7)
1	51 745 (21.4)	8358 (19.7)	27 848 (25.0)	12 773 (23.5)	27 908 (15.6)	3463 (14.5)	16 370 (20.1)	3830 (19.5)
2	15 867 (6.5)	2567 (6.0)	10 437 (9.4)	4893 (9.0)	6457 (3.6)	804 (3.4)	4943 (6.1)	1270 (6.5)
≥ 3	8895 (3.7)	1642 (3.9)	7341 (6.6)	3614 (6.7)	3263 (1.8)	507 (2.1)	2597 (3.2)	676 (3.4)
**Chronic disease**^**f**^, **n (%)**								
Yes	3146 (1.3)	1088 (2.6)	4746 (4.3)	2813 (5.2)	9922 (5.5)	1683 (7.1)	2564 (3.1)	811 (4.1)
**MMR vaccine**^**g**^, **n (%)**								
Yes	209 330 (86.4)	18 402 (43.3)	–	–	175 418 (98.0)	18 665 (78.3)	–	–
**DTaP/DTaP-IPV booster**^**h**^, **n (%)**								
Yes	–	–	38 175 (34.2)	16 689 (30.8)	15 673 (8.8)	2025 (8.5)	14 739 (18.1)	2411 (12.2)
**Influenza vaccine**^**i**^, **n (%)**								
Yes	–	–	15 201 (13.6)	4068 (7.5)	–	–	–	–
**Vaccine outside national immunization program**^**j**^, **n (%)**								
Yes	17 081 (7.0)	3334 (7.8)	17 381 (15.6)	6462 (11.9)	17 233 (9.6)	1757 (7.4)	–	–

HPV: human papillomavirus; SD: standard deviation; MMR: measles, mumps, rubella; DTaP: diphthetria, tetanus, acellular pertussis; DTaP-IPV: diphtheria, tetanus, acellular pertussis, inactivated polio

^a^ Vaccinated with HPV vaccine (at least one dose) during follow-up

^b^ Not vaccinated with HPV vaccine by the end of follow-up

^c^ Girls were categorized according to information from the year of their 10th birthday

^d^ Number of infectious disease hospitalizations in the pre-baseline period (9–11 years in Denmark, Finland, Norway; 8–10 years in Sweden)

^e^ Number of antibiotic treatment episodes in the pre-baseline period (9–11 years in Denmark, Finland, Norway; 8–10 years in Sweden)

^f^ Registered with a chronic disease diagnosis at any time between start of pre-baseline period (9 years in Denmark, Finland, Norway; 8 years in Sweden) and end of follow-up. Based on inpatient and outpatient hospital contacts

^g^ Vaccinated with MMR vaccine between age 11 years (Denmark)/10 years (Norway) and end of follow-up. MMR vaccine was not offered to girls in the study population in Finland and Sweden during the study period

^h^ Vaccinated with DTaP booster (Finland, Sweden) or DTaP-IPV booster (Norway) during follow-up. DTaP/DTaP-IPV booster was not offered to girls in the study population in Denmark during the study period

^i^ Vaccinated with influenza vaccine between age 11 years and end of follow-up. In Finland, girls in the study population may have been entitled to free-of-charge influenza vaccine if they belonged to a risk group or were close to a person susceptible to serious influenza

^j^ Vaccinated during follow-up with any vaccine not offered through the national immunization program to girls aged 11–14 years during the study period. Influenza vaccine was included among the non-program vaccines in Denmark and Norway, but not in Finland. In Sweden, non-program vaccines are not registered in the National Vaccination Register

The number of infectious disease hospitalizations during follow-up was 4446 in Denmark, 1404 in Finland, 2782 in Norway, and 1148 in Sweden, corresponding to incidence rates (per 10 000 person years) of 44.1, 35.7, 37.1, and 28.5, respectively (Supplementary Table 7).

In all countries, receipt of at least one dose of HPV vaccine was associated with decreased risk of infectious disease hospitalization. The unadjusted HR (95% CI) comparing vaccinated with unvaccinated person time was 0.59 (0.53, 0.67) in Denmark, 0.64 (0.55, 0.74) in Finland, 0.61 (0.52, 0.71) in Norway, and 0.52 (0.43, 0.63) in Sweden (Table 3). The associations were attenuated by covariate adjustment. The adjusted HR (95% CI) was 0.81 (0.72, 0.90) in Denmark, 0.69 (0.60, 0.80) in Finland, 0.76 (0.66, 0.88) in Norway, and 0.59 (0.49, 0.71) in Sweden.

**Table 3:** Hazard ratios of infectious disease hospitalization according to HPV vaccination status for girls in Denmark, Finland, Norway, and Sweden

	Numer of cases	Person years	Incidence rate per 10 000 person years (95% CI)	Unadjusted HR (95% CI)^a^	Adjusted HR (95% CI)^b^
**Denmark,** *n* ** = 284 824**					
Unvaccinated^c^	2070	426 464	48.5 (46.5, 50.7)	1 (Ref)	1 (Ref)
Vaccinated^d^	2376	581 752	40.8 (39.2, 42.5)	0.59 (0.53, 0.67)	0.81 (0.72, 0.90)
**Finland**,*n*** = 165 707**					
Unvaccinated^c^	849	212 795	39.9 (37.3, 42.7)	1 (Ref)	1 (Ref)
Vaccinated^d^	555	180 491	30.7 (28.3, 33.4)	0.64 (0.55, 0.74)	0.69 (0.60, 0.80)
**Norway**,*n*** = 202 774**					
Unvaccinated^c^	1348	316 647	42.6 (40.4, 44.9)	1 (Ref)	1 (Ref)
Vaccinated^d^	1434	432 803	33.1 (31.5, 34.9)	0.61 (0.52, 0.71)	0.76 (0.66, 0.88)
**Sweden**,*n*** = 101 153**					
Unvaccinated^c^	724	206 129	35.1 (32.7, 37.8)	1 (Ref)	1 (Ref)
Vaccinated^d^	424	196 910	21.5 (19.6, 23.7)	0.52 (0.43, 0.63)	0.59 (0.49, 0.71)

HPV: human papillomavirus; CI: confidence interval; HR: hazard ratio

^a^ Stratified Cox model with birth year as strata and age in days as the underlying time scale

^b^ Adjusted for household income, number of children in the household, having a single parent, maternal educational level, immigrant background, previous infectious disease hospitalization, number of previous antibiotic treatment episodes, receipt of diphtheria, tetanus acellular pertussis booster (Finland, Sweden only), receipt of diphtheria, tetanus, acellular pertussis, inactivated polio booster (Norway only), receipt of measles, mumps, rubella vaccine (Denmark, Norway only), receipt of influenza vaccine (Finland only), receipt of vaccine not offered through the national immunization program to girls aged 11–14 years (Denmark, Finland, Norway only), presence of chronic disease, and season

^c^ Girls were considered unvaccinated from start of follow-up until receipt of the first dose of HPV vaccine

^d^ Girls were considered vaccinated from receipt of the first dose of HPV vaccine until the end of follow-up

Receipt of HPV vaccine was also associated with decreased risk of hospitalization with respiratory tract infections, the secondary outcome (Supplementary Table 8). The associations tended to be slightly stronger than for infectious disease hospitalization overall.

The results among girls without immigrant background and among Danish and Norwegian girls offered Gardasil^®^ were very similar to the main results (Supplementary Tables 9 and 10, respectively).

**Fig. 2:**
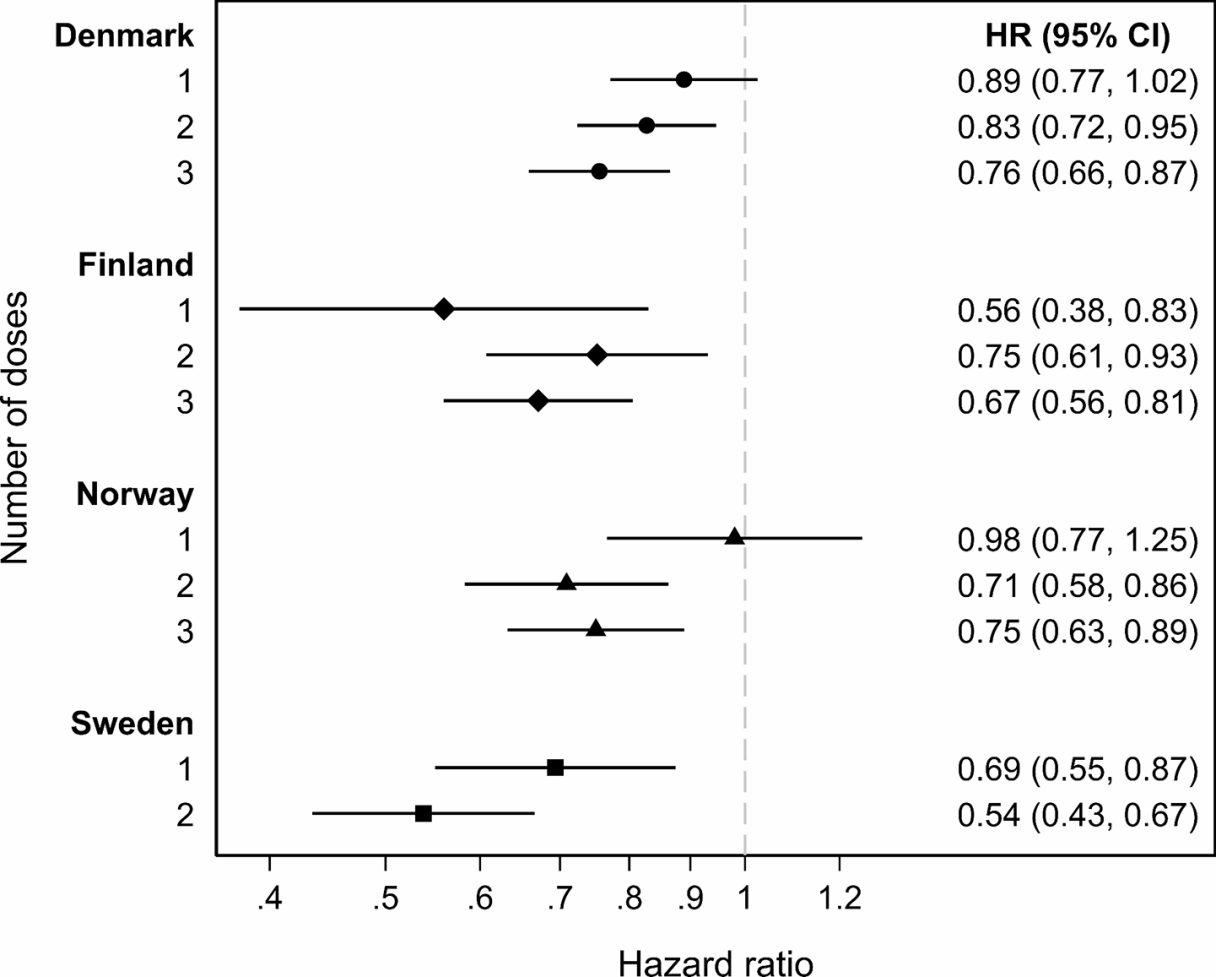
Adjusted hazard ratios of infectious disease hospitalization according to number of HPV vaccine doses as compared with unvaccinated person time. Horizontal bars indicate 95% confidence intervals. HRs and CIs were estimated using a stratified Cox model (birth year as strata) and attained age as time scale. Number of doses was a time-dependent variable updated at receipt of each dose. The model was adjusted for household income, number of children in the household, having a single parent, maternal educational level, immigrant background, previous infectious disease hospitalizations, number of previous antibiotic treatment episodes, presence of chronic disease, season, receipt of diphtheria, tetanus, acellular pertussis booster (Finland, Sweden only), receipt of diphtheria, tetanus, acellular pertussis, inactivated polio booster (Norway only), receipt of measles, mumps, rubella vaccine (Denmark, Norway only), receipt of influenza vaccine (Finland only), receipt of vaccine not offered through the national immunization program to girls aged 11–14 years (Denmark, Finland, Norway only)

Compared with being unvaccinated, having received only 1 dose of HPV vaccine was associated with decreased risk of infectious disease hospitalization in all countries except Norway: adjusted HR (95% CI) was 0.89 (0.77, 1.02) in Denmark, 0.56 (0.38, 0.83) in Finland, 0.98 (0.77, 1.25) in Norway, and 0.69 (0.55, 0.87) in Sweden (Table 4; Fig. 2). In the countries offering 3 doses (Denmark, Finland, Norway), having received 2 doses was associated with lower risk than being unvaccinated, with adjusted HRs ranging from 0.71 to 0.83. In all countries, having completed the vaccination series was associated with decreased risk: adjusted HR (95% CI) was 0.76 (0.66, 0.87) in Denmark, 0.67 (0.56, 0.81) in Finland, 0.75 (0.63, 0.89) in Norway, and 0.54 (0.43, 0.67) in Sweden. Generally, the adjusted HRs for each dose did not differ with time since vaccination, and in almost all time intervals we observed HRs below 1 (Supplementary Table 11). However, in Norway, a tendency towards increased risk was observed 90–180 days after dose 1, HR = 1.64 (95% CI 0.84, 3.22), and 181–365 days after dose 1, HR = 1.47 (95% CI 0.53, 4.08), but the estimates were imprecise with wide confidence intervals, as they were based on only 13 and 8 cases, respectively. In addition, a small increase was observed 181–365 days after dose 1 in Denmark, adjusted HR = 1.09 (95% CI 0.83, 1.43).

**Table 4:** Hazard ratios of infectious disease hospitalization according to number of HPV vaccine doses for girls in Denmark, Finland, Norway, and Sweden

	Numer of cases	Person years	Incidence rate per 10 000 person years (95% CI)	Unadjusted HR (95% CI)^a^	Adjusted HR (95% CI)^b^
**Denmark,***n* = 253 450^c^					
**Number of doses**^d^					
0	1842	365 025	50.5 (48.2, 52.8)	1 (Ref)	1 (Ref)
1	373	88 931	41.9 (37.9, 46.4)	0.72 (0.63, 0.83)	0.89 (0.77, 1.02)
2	613	152 702	40.1 (37.1, 43.5)	0.62 (0.54, 0.70)	0.83 (0.72, 0.95)
3	1322	312 595	42.3 (40.1, 44.6)	0.51 (0.45, 0.59)	0.76 (0.66, 0.87)
**Finland**,*n*** = 138 261**^**c**^					
**Number of doses**^**d**^					
0	698	166 824	41.8 (38.8, 45.1)	1 (Ref)	1 (Ref)
1	26	11 538	22.5 (15.3, 33.1)	0.53 (0.36, 0.79)	0.56 (0.38, 0.83)
2	134	44 283	30.3 (25.5, 35.8)	0.68 (0.55, 0.85)	0.75 (0.61, 0.93)
3	327	100 781	32.4 (29.1, 36.2)	0.61 (0.51, 0.73)	0.67 (0.56, 0.81)
**Norway**,*n*** = 173 473**^**c**^					
**Number of doses**^**d**^					
0	1219	276 432	44.1 (41.7, 46.6)	1 (Ref)	1 (Ref)
1	138	33 464	41.2 (34.9, 48.7)	0.94 (0.74, 1.20)	0.98 (0.77, 1.25)
2	203	64 779	31.3 (27.3, 36.0)	0.66 (0.54, 0.81)	0.71 (0.58, 0.86)
3	1007	301 092	33.4 (31.4, 35.6)	0.54 (0.45, 0.64)	0.75 (0.63, 0.89)
**Sweden**,*n*** = 101 153**^**e**^					
**Number of doses**^**d**^					
0	724	206 129	35.1 (32.7, 37.8)	1 (Ref)	1 (Ref)
1	129	52 346	24.6 (20.7, 29.3)	0.66 (0.52, 0.83)	0.69 (0.55, 0.87)
2	283	140 419	20.2 (17.9, 22.6)	0.45 (0.36, 0.57)	0.54 (0.43, 0.67)

HPV: human papillomavirus; CI: confidence interval; HR: hazard ratio

^a^ Stratified Cox model with birth year as strata and age in days as the underlying time scale

^b^ Adjusted for household income, number of children in the household, having a single parent, maternal educational level, immigrant background, previous infectious disease hospitalization, number of previous antibiotic treatment episodes, receipt of diphtheria, tetanus acellular pertussis booster (Finland, Sweden only), receipt of diphtheria, tetanus, acellular pertussis, inactivated polio booster (Norway only), receipt of measles, mumps, rubella vaccine (Denmark, Norway only), receipt of influenza vaccine (Finland only), receipt of vaccine not offered through the national immunization program to girls aged 11–14 years (Denmark, Finland, Norway only), presence of chronic disease, and season

^c^ Analyses restricted to birth cohorts eligible for three-dose schedule (1996–2003 in Denmark, 1998–2003 in Finland, 1999–2004 in Norway).Girls were censored at receipt of the first dose of HPV vaccine if this occurred after the two-dose schedule was implemented (February 1, 2016, in Denmark; start of school year 2016/2017 in Finland; start of school year 2017/2018 in Norway)

^d^ Girls were considered unvaccinated (0 doses) until receipt of the first dose of HPV vaccine. Number of doses was updated at receipt of each HPV vaccine dose

^e^ Girls were censored at receipt of the third dose, since the birth cohorts included in the study population were eligible for two-dose schedule

The additional analysis in Denmark included 59 248 girls (29.7%) who received the first dose of HPV vaccine after MMR vaccine (reference category) and 140 017 girls (70.3%) who received MMR vaccine and the first dose of HPV vaccine together. The two groups had similar risk of infectious disease hospitalization, adjusted HR = 1.01 (95% CI 0.90, 1.14) (Supplementary Table 12).

## Discussion

In this nationwide register-based cohort study, including nearly 800 000 girls from Denmark, Finland, Norway, and Sweden, we found that having received at least one dose of HPV vaccine was associated with a decreased risk of infectious disease hospitalization. In Denmark, Finland, and Sweden, being vaccinated was associated with decreased risk, regardless of number of doses. However, in Norway, girls who had received 2 or 3 doses had lower risks than unvaccinated girls, whereas no difference was observed between girls who had received only one dose and unvaccinated girls.

The strengths of our study pertain to the use of individual-level information from national registries in four Nordic countries, enabling complete follow-up of entire birth cohorts of girls offered HPV vaccine. The mandatory reporting to the registries ensures high-quality information on the outcome and exposures. Information in the patient registers is collected and recorded independently of the other registers, thus potential misclassification is most likely non-differential. The Nordic countries have tax funded universal health care; the vaccines in the NCIPs are offered free of charge, and inpatient hospital visits are free for children. The many similarities between the four countries allowed us to make meaningful comparisons of country-specific results and facilitated the interpretation and discussion on differences in effect estimates. The data was harmonized according to a common data model, and the statistical analyses were performed using identical syntax in each country, which further enhanced the comparability.

Chronic conditions and acute illness that can cause adverse health outcomes, may lead to delayed or missed vaccinations. In observational studies of non-specific vaccine effects, failure to properly control for such health-related factors will lead to a bias in the direction of a beneficial effect of the vaccine (healthy vaccinee bias). We adjusted for several potential confounders related to the participants’ health, such as presence of chronic disease, previous hospitalization, and previous antibiotic treatment episodes, which may serve as proxies for an underlying increased susceptibility to infections. Adjustment attenuated the associations, but vaccination was still associated with decreased risk in all countries. This suggests that healthy vaccinee bias was reduced. However, complete adjustment for all relevant health-related factors is not feasible. Such factors are complex, vary continuously over time, and may need to be measured from birth. Thus, healthy vaccinee bias cannot be entirely eliminated, and is probably the main explanation for the negative associations we observed. A limitation of our study is the lack of data from primary health care, which would have given us more extensive information on the participants underlying health, and potentially could have been used to further reduce the healthy vaccinee bias. Moreover, presence of chronic disease was based on hospital contacts from 8 years (in Sweden) or 9 years (in Denmark, Finland, and Norway) and onwards. Hospital contacts at younger ages might also be relevant. However, individual-level data on hospital contacts was not available until 2008 in Norway. This precluded the use of diagnoses from more than 2 years prior to baseline (the date the girls turned 11 years) for the oldest birth cohort. For consistency across birth cohorts and countries, the duration of the pre-baseline period was therefore set to 2 years. As a consequence, some misclassification of girls with a chronic disease is inevitable and this may have contributed to the healthy vaccinee bias. Finally, we had no information on common colds, fevers, or other acute illness episodes that do not lead to a doctor’s visit but are still considered contradictory to vaccination.

Healthy vaccinee bias might not solely explain the lower risk observed among HPV vaccinated girls. Even though causal conclusions cannot be drawn from this observational study, we cannot rule out that HPV vaccine may have beneficial non-specific effects. Moreover, vaccine-preventable diseases were included in the outcome since HPV vaccine could potentially have non-specific effects on infections preventable by other vaccines. We did not account for vaccines received before baseline in our analyses (with the exception of MMR vaccine in Norway). This was primarily determined by data availability. The vaccination registers in Finland and Sweden were established in 2009 and 2013, respectively. Thus, information on vaccines received during the first years of life was not available for any of the girls from Finland (born 1998–2004) and Sweden (born 2003–2004). Moreover, complete vaccination history could not be obtained for the many Danish and Norwegian girls who had not been residents continuously from birth to start of follow-up. Our inclusion and exclusion criteria ensured that we had complete information on vaccinations received from baseline until end of follow-up for all the girls included in our study. Residual confounding due to lack of adjustment for vaccines received before baseline may partly explain why we observed lower risk among the HPV vaccinated girls, since the HPV vaccinated girls may be more likely to have received other vaccines and therefore better protected against vaccine-preventable diseases. However, the number of girls diagnosed with a vaccine-preventable disease was low. The Nordic countries have very high coverage of all the vaccines offered through the NCIPs, and many diseases, such as polio, diphtheria, measles, mumps, and rubella have been practically eliminated. Other vaccine-preventable diseases, not covered by the vaccines in the NCIPs, are also rare in the age groups included in our study. The number of girls hospitalized with influenza, the most frequently diagnosed vaccine-preventable disease, was 58 in Denmark, 29 in Finland, 62 in Norway, and 17 in Sweden, corresponding to 1–2% of hospitalizations included in the outcome. In addition, we adjusted for receipt of other vaccines, including vaccine against influenza, from baseline and onwards. Hence, the residual confounding due to incomplete adjustment for vaccination history is likely modest.

The HPV vaccine uptake was highest and increased more rapidly with age in Norway than in the other countries [26]. Thus, at each age, the group of girls who remained unvaccinated was smaller and likely less representative in Norway. The groups being compared may therefore be more unbalanced in terms of unmeasured confounders. In that case, we would expect healthy vaccinee bias to be most pronounced in Norway. However, the association was slightly weaker in Norway (HR = 0.76) than in e.g., Finland (HR = 0.69), where HPV vaccine uptake was lowest.

Healthy vaccinee bias may differ with time since vaccination. At the time of vaccination, children will tend to be free from acute infections that may subsequently lead to hospitalization. Consequently, healthy vaccinee bias might be most pronounced shortly after vaccination. We did not observe a consistent pattern of lower risk in the first 1–14 days after vaccination compared to later. However, there were few cases in this short interval, and the precision of the effect estimates was low. On the other hand, healthy vaccinee bias might not be present during later time intervals when girls are scheduled for the next dose. According to the recommended 3-dose schedule used in Denmark and Norway, dose 2 should be given 2 months after dose 1. Thus, girls who contribute person time in categories more than 90 days after vaccination with dose 1 have for some reason not received dose 2 according to the schedule. The delay may have been caused by health-related factors, e.g., an acute illness. This might explain why we found effect estimates above 1 more than 90 days after the first dose in Denmark and Norway.

We did not have information on parents’ health-consciousness or attitudes towards vaccines. Such factors may be more important predictors of HPV vaccination in Denmark, where parents need to book a vaccination appointment with their general practitioner, than in the other countries, where vaccination programs are school-based. Consequently, the bias due to lack of adjustment for these factors might be more pronounced in Denmark. Although it is difficult to predict the direction of the bias, daughters of health-conscious parents might be more likely both to receive HPV vaccine *and* to seek medical care. If so, the bias would be directed towards a deleterious effect of the vaccine, opposite of healthy vaccine bias, which would explain why the weakest association was observed in Denmark.

In Denmark, the risk of hospitalization was very similar among girls who received MMR and HPV vaccines together and those who received HPV vaccine after MMR vaccine. Since all girls included in this analysis had received both MMR and HPV vaccines, although in different sequence, we would expect the groups being compared to be quite similar with respect to unmeasured confounders. Nevertheless, underlying health issues could have caused parents and general practitioners to become hesitant toward co-administration of vaccines, but this would result in a bias towards a beneficial effect of simultaneous receipt of MMR and HPV vaccine. Thus, our results do not support that receiving MMR and HPV vaccines together was more beneficial than receiving HPV vaccine alone.

We limited the outcome in our study to hospitalizations with at least one overnight stay, thereby including mainly the more severe infections. Registration of the less severe infections may be more influenced by health care organization and coding practices, which may vary more between countries. Thus, the outcome is more comparable across countries than if we had included outpatient contacts and inpatient contact without overnight stay [30]. Nevertheless, the infectious disease hospitalization rate was considerably higher in Denmark than in Sweden, with intermediate rates observed in Finland and Norway. The hospitalization rate is determined by a complex set of predictors. That the rate differed across countries may indicate that the importance of these predictors may vary. If so, the residual confounding due to insufficient control for such predictors, may be more substantial in some countries than in others, which may have contributed to the differences in the effect estimates.

The results from our study are in accordance with previous studies. Hviid et al. conducted a self-controlled case series analysis, based on the Danish National Patient Registry [8]. Small decreased risks of hospitalization due to any infection, upper respiratory tract infection, lower respiratory tract infection, gastrointestinal infection, and other infection were observed in the main risk period 0–90 days after HPV vaccination. In a study using health care records from the United States, women and girls who had received HPV vaccine less than two years previously had lower risk of COVID-19 compared to unvaccinated, propensity-score matched controls [9]. Finally, hospital contact due to pneumonia was used as a negative control outcome in a nationwide registry-based study from Denmark that evaluated the association between HPV vaccination and hospital contacts due to pain, severe fatigue, or circulatory symptoms [31]. HPV vaccination was associated with a decreased risk of pneumonia both in a cohort analysis and a self-controlled case series analysis.

## Conclusion

We found no evidence that HPV vaccination increases the risk of infectious disease hospitalization in this nationwide cohort study of girls from four Nordic countries. Receipt of HPV vaccine was consistently associated with a decreased risk of infectious disease hospitalization, suggesting that healthy vaccinee bias may not have been entirely eliminated in the analyses. Yet, our results are reassuring, and do not support that the non-live HPV vaccine has deleterious non-specific effects.

## Electronic supplementary material

Below is the link to the electronic supplementary material.

Supplementary Material 1
