# Supplementary material for: Infectious disease hospitalization after receipt of human papillomavirus vaccine: a nationwide register-based cohort study among Danish, Finnish, Norwegian, and Swedish girls
Source: Eur J Epidemiol. 2025 Jan 6;40(1):81–93. doi: 10.1007/s10654-024-01197-3 (PMC11799007; doi:10.1007/s10654-024-01197-3)
Supplement: Supplementary file 1 — Supplementary Material 1 [file 10654_2024_1197_MOESM1_ESM.pdf]

Supplementary material:

Infectious disease hospitalization after receipt of human papillomavirus vaccine: A nationwide register-based cohort study among Danish, Finnish, Norwegian, and Swedish girls

Ida Laake, Berit Feiring, Lise Gehrt, Hélène Englund, Mika Lahdenkari, Signe Sørup, Heta Nieminen, Lill Trogstad

Corresponding author: Ida Laake, ida.laake@fhi.no

Table of Contents

Supplementary table 1: Vaccines offered through the national immunization program to girls between ages 4 and 15 years in Denmark 2

Supplementary table 2: Vaccines offered through the national immunization program to girls between ages 4 and 15 years in Finland 3

Supplementary table 3: Vaccines offered through the national immunization program to girls between ages 4 and 15 years in Norway 4

Supplementary table 4: Vaccines offered through the national immunization program to girls between ages 4 and 15 years in Sweden 5

Supplementary Table 5: ICD-10 codes used to define infectious disease hospitalization 6

Supplementary table 6: Description of registry-based covariates 8

Supplementary figure 1: Variable assessment periods in Denmark, Finland, and Norway. 12

Supplementary figure 2: Variable assessment periods in Sweden. 13

Supplementary table 7: Age-specific incidence rates of infectious disease hospitalization 14

Supplementary table 8: Hazard ratios of hospitalization with respiratory tract infection according to HPV vaccination status for girls in Denmark, Finland, Norway, and Sweden 15

Supplementary table 9: Hazard ratios of infectious disease hospitalization according to HPV vaccination status among girls without immigrant background 16

Supplementary table 10: Hazard ratios of infectious disease hospitalization according to HPV vaccination status and time since vaccination for girls in Denmark and Norway who were offered Gardasil 17

Supplementary table 11: Hazard ratios of infectious disease hospitalization according to number of HPV vaccine doses and time since vaccination for girls in Denmark, Finland, Norway, and Sweden 18

Supplementary table 12: Hazard ratios of infectious disease hospitalization comparing girls who received MMR vaccine together with the first dose of HPV vaccine to girls who received MMR vaccine before the first dose of HPV vaccine 22

Supplementary table 1: **Vaccines offered through the national immunization program to girls between ages 4 and 15 years in Denmark**

Birth cohort	4 years	5 years	12 years
**1996–1997**	OPV	DT booster	MMR, HPV[a]
**1998**	OPV	DT[b] or DTaP booster[c]	MMR, HPV
**1999**[d]	OPV[d]	DTaP booster	MMR, HPV
**1999**[e]**–Mar 2004**[f]		DTaP-IPV booster[e]	MMR, HPV
**Apr 2004**[f]**–2005**	MMR[f]	DTaP-IPV booster	HPV

OPV: Oral polio vaccine; DT: diphtheria, tetanus; MMR: measles, mumps, rubella; HPV: human papillomavirus; DTaP: diphtheria, tetanus, acellular pertussis; DTaP-IPV: diphtheria, tetanus, acellular pertussis, inactivated polio.

[a] Since January 1, 2009, HPV vaccine has been offered to girls born in 1996 and later [1, 2].

[b] DT booster offered until August 31, 2003 [3].

[c] DTaP booster offered Sept 1, 2003–June 30, 2004 [3].

[d] Phase-out of OPV from July 1, 2001 [4]. Until August 31, 2003, OPV was offered in three-dose schedule (at 2, 3, and 4 years) to children who had received the first dose prior to July 1, 2001 (in principle, children born prior to July 1, 1999) [5].

[e] Children turning 5 years as of July 1, 2004, (born July 1, 1999 and later) were not offered OPV [4]. DTaP-IPV booster offered from July 1, 2004 [3].

[f] MMR vaccine offered at age 4 years from April 1, 2008 (children born April 1, 2004, and later)[6]. Children born prior to April 1, 2004, were still offered MMR vaccine at age 12 years.

Supplementary table 2: Vaccines offered through the national immunization program to girls between ages 4 and 15 years in Finland

Birth cohort	4 years	6 years	6th grade (12 years)	7th–9th grade (13–15 years)	14–15 years
**1998–2000**		MMR, DTaP + IPV or DTaP-IPV booster[a]		HPV[b]	DTaP booster[c]
**2001–2003**		MMR, DTaP + IPV or DTaP-IPV booster	HPV		DTaP booster
**2004–2005**	DTaP-IPV booster[d]	MMR	HPV		DTaP booster

MMR: measles, mumps, rubella; DTaP: diphtheria, tetanus, acellular pertussis; IPV: inactivated polio vaccine; DTaP-IPV: diphtheria, tetanus, acellular pertussis, inactivated polio; HPV: human papillomavirus.

[a] DTaP or DTaP-IPV booster was offered at age 6 years from 2003 to 2010 [7].

[b] Since November 2013, HPV vaccine has been offered to girls born in 1998 and later [7, 8]. During the first 2 years, the oldest birth cohorts were offered the vaccine in grades 7–9.

[c] Since 2011, DTaP booster has been offered at age 14–15 years of age to those born in 1997 and later [9].

[d] DTaP-IPV booster has been offered at age 4 years since 2008 [7].

Supplementary table 3: Vaccines offered through the national immunization program to girls between ages 4 and 15 years in Norway

Birth cohort	2nd grade (7 years)	6th grade (11 years)	7th grade (12 years)	10th grade (15 years)
**1997**	IPV booster[a]	DT booster[b], MMR	HPV[c]	IPV booster[d]
**1998–2005**	DTaP-IPV booster[e]	MMR	HPV	DTaP-IPV booster[f]

IPV: inactivated polio vaccine; DT: diphtheria, tetanus; MMR: measles, mumps, rubella; HPV: human papillomavirus; DTaP-IPV: diphtheria, tetanus, acellular pertussis, inactivated polio.

[a] IPV booster offered at age 6-8 years until 2006 [10].

[b] DT booster offered in 6th grade until the school year 2008/2009 [10].

[c] Since the school year 2009/2010, HPV vaccine has been offered to girls born in 1997 and later [10].

[d] IPV booster offered in 10th grade to children born in 1997 and before (until school year 2012/2013) [11]

[e] DTaP-IPV booster offered during 2nd grade to children born in 1998 and later [10].

[f] DTaP-IPV booster offered in 10th grade to children born in 1998 and later (from school year 2013/2014) [10].

Supplementary table 4: Vaccines offered through the national immunization program to girls between ages 4 and 15 years in Sweden

Birth cohort	5–6 years	1st or 2nd grade (6–8 years)	4th grade (10 years)	5th or 6th grade (10–12 years)	6th grade (12 years)	8th or 9th grade (14–16 years)
**1999–2001**	IPV booster[a]		DTaP booster[b]	HPV[c]	MMR[d]	
**2002–2005**	DTaP-IPV booster[e]	MMR[f]		HPV		DTaP booster[g]

IPV: inactivated polio vaccine; DTaP: diphtheria, tetanus, acellular pertussis; HPV: human papillomavirus; MMR: measles, mumps, rubella; DTaP-IPV: diphtheria, tetanus, acellular pertussis, inactivated polio.

[a] IPV booster offered at age 5–6 years to children born in 2001 and before [12].

[b] DTaP booster offered in 4th grade to children born in 1995–2001 (until school year 2011/2012) [12].

[c] Since January 2012, HPV vaccine has been to girls born in 1999 and later [12, 13].

[d] MMR vaccine offered at age 12 years (in 6th grade) to children born in 2001 and before [12].

[e] DTaP-IPV booster offered at age 5–6 years to all children born in 2002 and later [12].

[f] MMR offered in 1st or 2nd grade to children born in 2002 and later (since school year 2009/2010) [12].

[g] DTaP booster offered in 8th or 9th grade to children born in 2002 and later (since school year 2016/2017) [12].

Supplementary Table 5: ICD-10 codes used to define infectious disease hospitalization[a]

Group of infections	Diagnosis	ICD10–codes
**Respiratory tract infections**	Peritonsillar abscess	J36
	Infections in the ear	H65–H67, H68.0, H70, H73.0
	Laryngitis	A36.2, J04-J05, J37.0
	Nasopharyngitis	A36.1, J00
	Pharyngitis	J02
	Sinusitis	J01.0-J01.1, J01.3-J01.9, J32
	Ethmoiditis	J01.2
	Tonsillitis	A36.0, J03, J35.0
	Influenza	J09–J11
	Pneumonia	A48.1, A70, J12–J18
	Other respiratory infections	A36.8–A36.9, A37, A42.0, J06, J20–J22, J40, J44.0, J85–J86
**Gastrointestinal infections**	Gastrointestinal infections	A00-A02.0, A02.2–A09.9, A42.1
**Other infections**	Acute lymphadenitis	L04
	Cellulitis and abscess	H60.0–H60.1, L02–L03
	Dermatophytosis and other superficial mycoses	B35–B36
	Erysipelas	A46
	Viral warts[b]	B07
	Other local infections of skin and subcutaneous tissue	A36.3, H60.2–H60.3, H60.8–H60.9, L00–L01, L08, L30.3
	Certain bacterial diseases	A20–A32.0, A32.8–A35.9, A38, A39.1, A39.3–A39.9, A42.2–A44.9, A48.0, A48.2–A49.9, A74.8–A74.9
	Cystitis	N30.0
	Hepatitis	B15–B19
	Infections of the circulatory system	I00–I01
	Infections of the eye	A71–A74.0, H04.0, H04.3, H04.4, H06.1, H10.0, H10.2–H10.3, H10.5–H10.9, H13.1
	Infections of the musculoskeletal system and connective tissue	M00–M01, M86
	Infections of the nervous system	G04–G07
	Infections of the urinary system	N34.0–N34.1, N39.0

	Meningitis	A32.1, A39.0, A87, B00.3–B00.4, B01.0–B01.1, B02.0–B02.1, B05.0–B05.1, B06.0, B26.1–B26.2, G00-G02, G03.9
	Mycoses	B37.0–B37.6, B37.8–B49.9
	Protozoal diseases	B50–B89
	Pyelonephritis	N10, N12
	Rickettsiosis	A75–A79
	Sepsis	A02.1, A32.7, A39.2, A40–A41, B37.7
	Spirochaetal disease	A65–A69
	Tuberculosis	A15–A19, K.93.0
	Bacterial, viral and other infectious agents as the cause of diseases classified to other chapters	B95-B98
	Unspecified infectious diseases	B99
	Viral Infections	A80–A86, A88-A89, A92–A99, B00.0–B00.2, B00.5–B00.9, B01.2–B01.9, B02.2–B04.9, B05.2–B05.9, B06.1–B06.9, B08–B09, B25–B26.0, B26.3–B34.9
	Sexually transmitted diseases	A50–A60, A63.8–A64.9, B20-B24
	Fever unspecified	R50

ICD-10: International Classification of Diseases, Tenth Revision.

[a] Definition of infectious disease was based on Gehrt et al. [14]. However, in the present, study, we did not include A63.0 Anogenital (venereal) warts in the outcome since these are caused by human papillomavirus types 6 and 11, which are targeted by the quadrivalent vaccine, Gardasil®.

[b] B07 is used exclusively for non-anogenital warts. These are caused by human papillomavirus types not targeted by vaccines.

Supplementary table 6: Description of registry-based covariates				
**Covariate**	**Description**	**Categorization**	**Countries where covariate was included in analyses**	**Time-dependent**
Household income	Household income quintile in the year of the girl's 10th birthday.	1 (lowest), 2, 3, 4, 5 (highest), or missing	All	No
Number of children in household	Number of children below 18 years in the household, including the girl herself, in the year of the girl's 10th birthday	1, 2, 3, ≥ 4, or missing	All	No
Single parent	Girls living with a single parent in the year of 10th birthday was categorized as having a single parent.	Yes, no, or missing	All	No
Maternal educational level	Mother's highest attained education in the year of the girl's 10th birthday.	Low, medium, high, or missing	All	No
Immigrant background	Girls without a parent born in the country where the girl was a citizen (or permanent resident) were categorized as having immigrant background.	Yes, no	All	No
Previous infectious disease hospitalization	Infectious disease hospitalization during pre-baseline period[a]. Hospitalizations with a date of discharge less than 14 days prior to start of follow-up were not included, to avoid counting hospitalizations that were a result of the same infection that later led to a hospitalization counted as an outcome.	Yes, no	All	No
Number of previous antibiotic treatment episodes	Number of antibiotic treatment episodes [15] during pre-baseline period[a]. Prescriptions with a date of redemption less than 14 days prior to start of follow-up were not included, to avoid	0, 1, 2, ≥ 3	All	No

	counting antibiotic treatments that were a result of the same infection that later led to a hospitalization counted as an outcome.			
Presence of chronic conditions	All hospital contacts (inpatient and outpatient) from the start of the pre-baseline period[a] until the end of follow-up were used to identify girls with a chronic disease, as defined by Kristensen et al. [16]. Date of onset was defined as first date of a registered diagnosis falling within the definition, or date of birth for girls with congenital conditions. Girls were classified as having a chronic disease from date of onset until end of follow-up. Girls with date of onset before start of follow were classified as having a chronic disease throughout follow-up.	Yes, no	All	Yes
DTaP booster	DTaP booster is offered in Finland and Sweden at age 14–15 and age 14–16 years, respectively. To define the variable, we used vaccinations received from baseline[b] and later. Girls were categorized as vaccinated from receipt of the DTaP booster until the end of follow-up. Girls who received DTaP booster before start of follow-up (but after baseline), were considered vaccinated throughout follow-up.	Yes, no	Finland, Sweden	Yes
DTaP-IPV booster	DTaP-IPV booster is offered at age 15 years in Norway. To define the variable, we used	Yes, no	Norway	Yes

	vaccinations received from baseline[b] or later. Girls were categorized as vaccinated from receipt of the DTaP-IPV booster until the end of follow-up.			
MMR vaccine	Revaccination with MMR vaccine was offered at age 12 years until 2016 in Denmark and is offered in 6th grade (around age 11 years) in Norway. To define the variable, we used vaccinations received from baseline (age 11 years) or later in Denmark. In Norway, we used vaccinations received at 10 years or later, since MMR vaccine may have been offered prior to the 11th birthday. Girls were categorized as vaccinated from receipt of MMR vaccine until the end of follow-up. Girls who received MMR vaccine before start of follow-up (but after age 11/10 years), were considered vaccinated throughout follow-up.	Yes, no	Denmark, Norway	Yes
Influenza vaccine	In Finland, annual free-of-charge influenza vaccine was offered to all children aged 6–36 months during the study period. Older children may have been entitled to free-of-charge vaccine if they belonged to a risk group or were close to a person susceptible to serious influenza. Receipt of influenza vaccine was quite common among the Finnish girls in our study (aged 11–14 years) and was therefore included as a covariate. To define the variable, we used	Yes, no	Finland	Yes

	vaccinations received from baseline (age 11 years) or later. Girls were categorized as vaccinated from receipt of influenza vaccine until the end of follow-up. Girls who received influenza vaccine before start of follow-up (but after age 11), were considered vaccinated throughout follow-up.			
Non-program vaccines	Receipt of vaccines not offered through the national immunization program to girls aged 11–14 years during the study period. Influenza vaccine was included among the non-program vaccines in Denmark and Norway, but not in Finland. In Sweden, non-program vaccines are not registered in the National Vaccination Register. To define the variable, we used vaccinations received from baseline (age 11 years) or later. Girls were categorized as vaccinated from receipt of the first non-program vaccine until the end of follow-up. Girls who received a non-program vaccine before start of follow-up (but after age 11), were considered vaccinated throughout follow-up.	Yes, no	Denmark, Finland, Norway	Yes

MMR: measles, mumps, rubella; DTaP: diphtheria, tetanus, acellular pertussis; DTaP-IPV: diphtheria, tetanus, acellular pertussis, inactivated polio.

[a] The pre-baseline period started at age 9 years and ended at age 11 years in Finland, Denmark, and Norway. In Sweden, the pre-baseline period started at age 8 years and ended at age 10 years.

[b] Baseline was defined as the date the girls turned 11 years in Denmark, Finland, Norway, and the date they turned 10 years in Sweden.

Supplementary figure 1: Variable assessment periods in Denmark, Finland, and Norway.

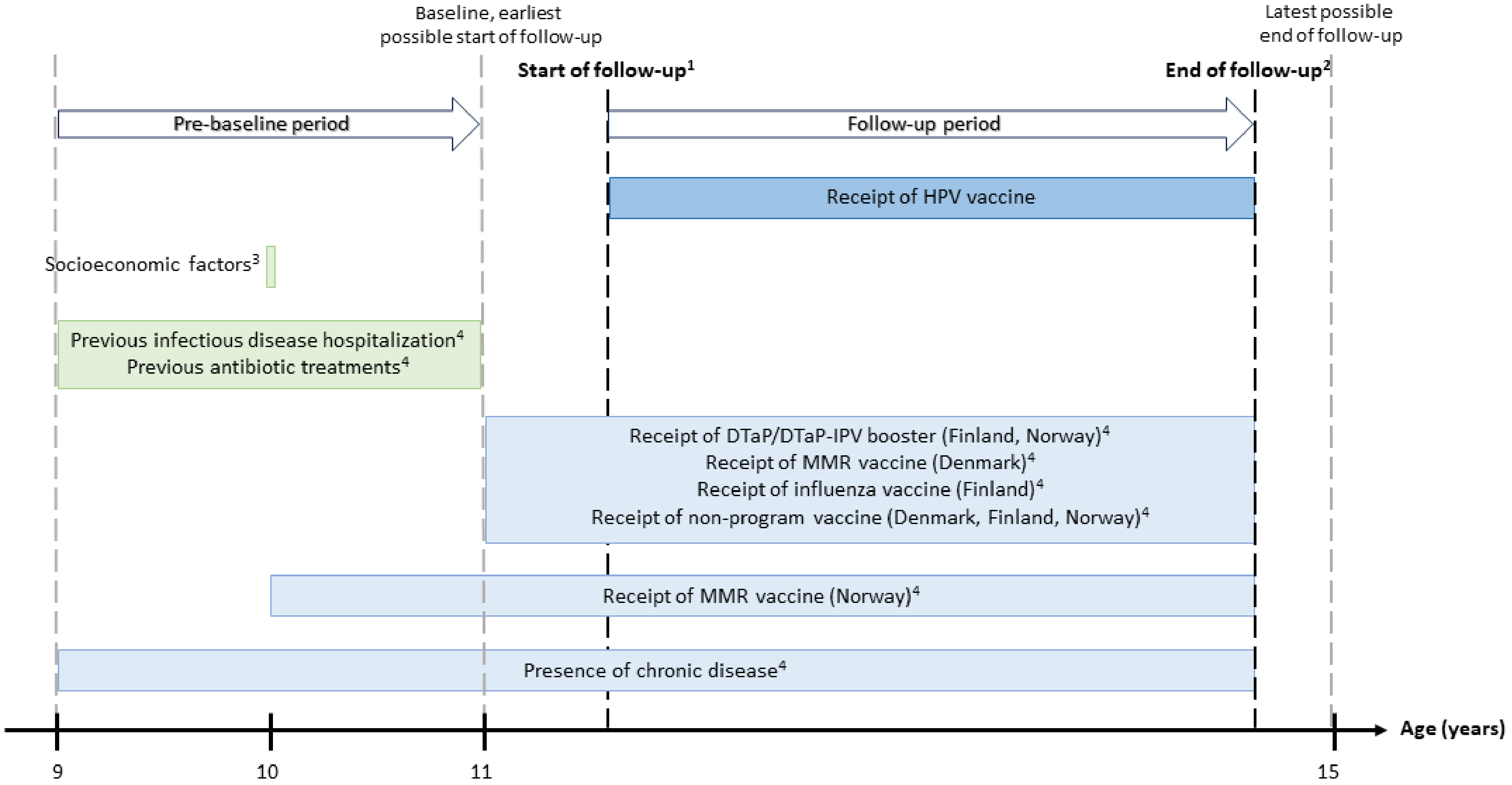

Note: The green boxes represent variables that are time-independent, i.e., the value of the variable does not change during follow-up. The blue boxes represent variables that are time-dependent, i.e., the value of the variable may be updated during follow-up. The darker blue box represents the main exposure (HPV vaccination).

HPV: human papillomavirus; DTaP: diphtheria, tetanus, acellular pertussis; DTaP-IPV: diphtheria, tetanus, acellular pertussis, inactivated polio; MMR: measles, mumps, rubella.

[1] 11th birthday or start of study period, whichever occurred last. Danish girls born 1996–1997 and Finnish girls born before Nov 1, 2002, were followed from start of study period, which occurred after these girls had turned 11 years old. Danish girls born 1998–2004; Finnish girls born Nov 1, 2002–Dec 31, 2004; and all Norwegian girls were followed from age 11 years.

[2] Death, emigration, 15h birthday, or end of study period, whichever occurred first. Danish girls born 1996–2002; Finnish girls born Nov 1, 1998–Dec 31, 2002; and Norwegian girls born 1999–2003 were followed until they turned 15 years (unless they died or emigrated). For girls born later, the end of the study period occurred before they turned 15 years.

[3] Household income, number of children in the household, having a single parent, maternal education. See Supplementary table 6 for further details.

[4] See Supplementary table 6 for further details.

Supplementary figure 2: Variable assessment periods in Sweden.

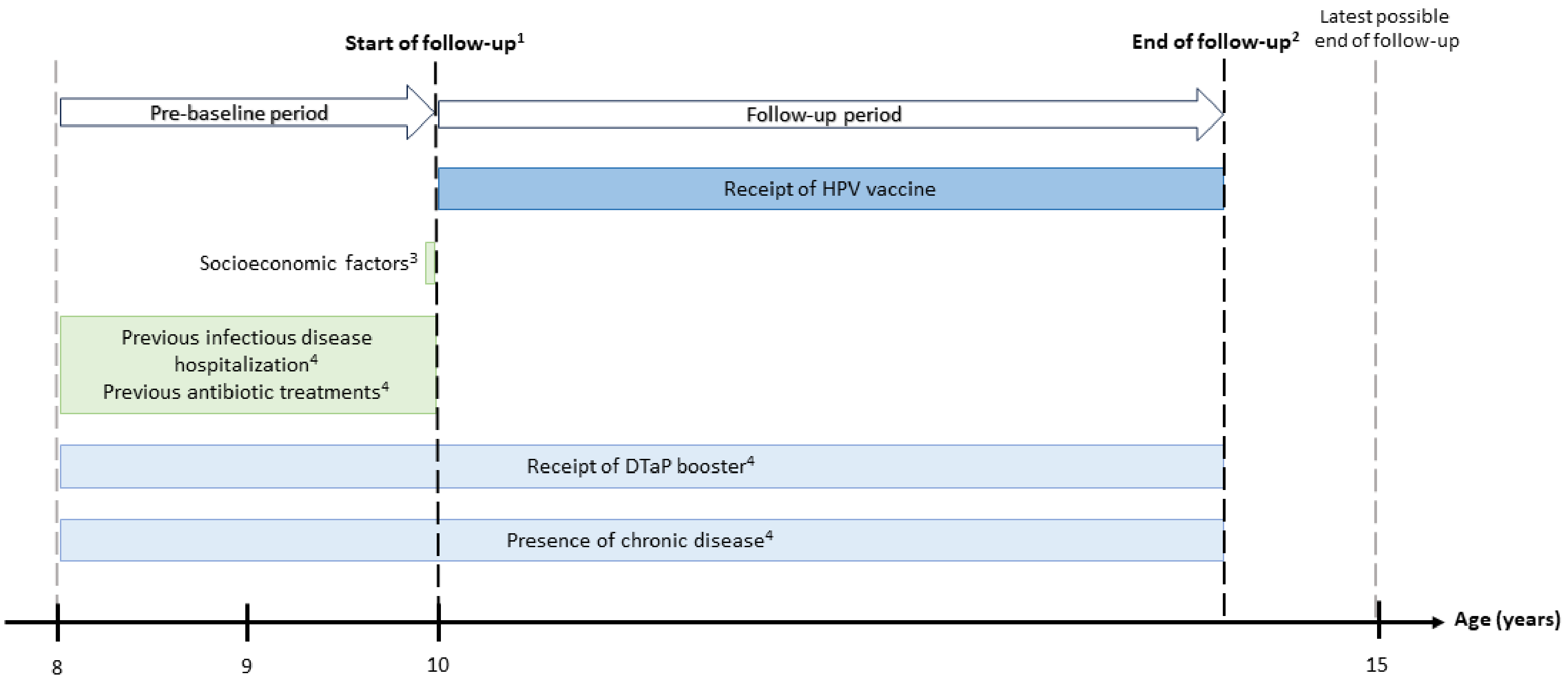

Note: The green boxes represent variables that are time-independent, i.e., the value of the variable does not change during follow-up. The blue boxes represent variables that are time-dependent, i.e., the value of the variable may be updated during follow-up. The darker blue box represents the main exposure (HPV vaccination).

HPV: human papillomavirus; DTaP: diphtheria, tetanus, acellular pertussis; DTaP-IPV: diphtheria, tetanus, acellular pertussis, inactivated polio; MMR: measles, mumps, rubella.

[1] All Swedish girls were followed from 10th birthday (baseline).

[2] Death, emigration, 15h birthday, or end of study period (Dec 31, 2017), whichever occurred first. Only girls born Jan 1, 2003, could be followed until their 15th birthday. For girls born later, end of the study period occurred before they turned 15 years.

[3] Household income, number of children in the household, having a single parent, maternal education. See Supplementary table 6 for further details.

[4] See Supplementary table 6 for further details.

Supplementary table 7: Age-specific incidence rates of infectious disease hospitalization

	DENMARK			FINLAND			NORWAY			SWEDEN		
**Age in years**	**Cases**	**Person years**	**Incidence rate per 10 000 person years (95% CI)**	**Cases**	**Person years**	**Incidence rate per 10 000 person years (95% CI)**	**Cases**	**Person years**	**Incidence rate per 10 000 person years (95% CI)**	**Cases**	**Person years**	**Incidence rate per 10 000 person years (95% CI)**
10										339	101 163	33.5 (30.1, 37.3)
11	1072	236 746	45.3 (42.6, 48.1)	218	72 316	30.1 (26.4, 34.4)	780	203 020	38.4 (35.8, 41.2)	265	100 701	26.3 (23.3, 29.7)
12	1044	268 013	39.0 (36.7, 41.4)	321	98 466	32.6 (29.2, 36.4)	685	201 934	33.9 (31.5, 36.6)	269	100 481	26.8 (23.8, 30.2)
13	1113	267 803	41.6 (39.2, 44.1)	317	111 553	28.4 (25.5, 31.7)	662	187 072	35.4 (32.8, 38.2)	205	75 567	27.1 (23.7, 31.1)
14	1217	235 656	51.6 (48.8, 54.6)	548	110 952	49.4 (45.4, 53.7)	655	157 425	41.6 (38.5, 44.9)	70	25 127	27.9 (22.0, 35.2)
**Total**	4446	1 008 217	44.1 (42.8, 45.4)	1404	393 287	35.7 (33.9, 37.6)	2782	749 450	37.1 (35.8, 38.5)	1148	403 038	28.5 (26.9, 30.2)

CI: confidence interval.

Supplementary table 8: Hazard ratios of hospitalization with respiratory tract infection according to HPV vaccination status for girls in Denmark, Finland, Norway, and Sweden

	Numer of cases	Person years	Incidence rate per 10 000 person years (95% CI)	Unadjusted HR (95% CI)[a]	Adjusted HR (95% CI)[b]
**DENMARK, n = 284 824**					
Unvaccinated[c]	913	426 464	21.4 (20.1, 22.8)	1 (Ref)	1 (Ref)
Vaccinated[d]	1043	581 752	17.9 (16.9, 19.1)	0.54 (0.46, 0.64)	0.75 (0.64, 0.88)
**FINLAND, n = 165 707**					
Unvaccinated[c]	427	212 795	20.1 (18.3, 22.1)	1 (Ref)	1 (Ref)
Vaccinated[d]	261	180 491	14.5 (12.8, 16.3)	0.60 (0.49, 0.73)	0.65 (0.53, 0.79)
**NORWAY, n = 202 774**					
Unvaccinated[c]	688	316 647	21.7 (20.2, 23.4)	1 (Ref)	1 (Ref)
Vaccinated[d]	709	432 803	16.4 (15.2, 17.6)	0.55 (0.45, 0.67)	0.64 (0.54, 0.77)
**SWEDEN, n = 101 153**					
Unvaccinated[c]	278	206 129	13.5 (12.0, 15.2)	1 (Ref)	1 (Ref)
Vaccinated[d]	174	196 910	8.8 (7.6, 10.3)	0.49 (0.37, 0.66)	0.53 (0.40, 0.71)

HPV: human papillomavirus; CI: confidence interval; HR: hazard ratio.

[a] Stratified Cox model with birth year as strata and age in days as the underlying time scale.

[b] Adjusted for household income, number of children in the household, having a single parent, maternal educational level, immigrant background, previous infectious disease hospitalization, number of previous antibiotic treatment episodes, receipt of diphtheria, tetanus, acellular pertussis booster (Finland, Sweden only), receipt of diphtheria, tetanus, acellular pertussis, inactivated polio booster (Norway only), receipt of measles, mumps, rubella vaccine (Denmark, Norway only), receipt of influenza vaccine (Finland only), receipt of vaccine not offered through the national immunization program to girls aged 11–14 years (Denmark, Finland, Norway only), presence of chronic disease, and season.

[c] Girls were considered unvaccinated from start of follow-up until receipt of the first dose of HPV vaccine.

[d] Girls were considered vaccinated from receipt of the first dose of HPV vaccine until the end of follow-up.

Supplementary table 9: Hazard ratios of infectious disease hospitalization according to HPV vaccination status among girls without immigrant background

	Numer of cases	Person years	Incidence rate per 10 000 person years (95% CI)	Unadjusted HR (95% CI)[a]	Adjusted HR (95% CI)[b]
**DENMARK, n = 257 413**					
Unvaccinated[c]	1866	384 477	48.5 (46.4, 50.8)	1 (Ref)	1 (Ref)
Vaccinated[d]	2172	527 805	41.2 (39.5, 42.9)	0.58 (0.52, 0.66)	0.80 (0.71, 0.90)
**FINLAND, n = 161 156**					
Unvaccinated[c]	816	205 579	39.7 (37.1, 42.5)	1 (Ref)	1 (Ref)
Vaccinated[d]	538	176 729	30.4 (28.0, 33.1)	0.63 (0.54, 0.72)	0.68 (0.59, 0.79)
**NORWAY, n = 178 688**					
Unvaccinated[c]	1198	278 237	43.1 (40.7, 45.6)	1 (Ref)	1 (Ref)
Vaccinated[d]	1296	385 544	33.6 (31.8, 35.5)	0.59 (0.50, 0.70)	0.75 (0.64, 0.87)
**SWEDEN, n = 83 593**					
Unvaccinated[c]	566	169 005	33.5 (30.8, 36.4)	1 (Ref)	1 (Ref)
Vaccinated[d]	366	165 332	22.1 (20.0, 24.5)	0.53 (0.42, 0.66)	0.59 (0.47, 0.73)

HPV: human papillomavirus; CI: confidence interval; HR: hazard ratio.

[a] Stratified Cox model with birth year as strata and age in days as the underlying time scale.

[b] Adjusted for household income, number of children in the household, having a single parent, maternal educational level, immigrant background, previous infectious disease hospitalization, number of previous antibiotic treatment episodes, receipt of diphtheria, tetanus, acellular pertussis booster (Finland, Sweden only), receipt of diphtheria, tetanus, acellular pertussis, inactivated polio booster (Norway only), receipt of measles, mumps, rubella vaccine (Denmark, Norway only), receipt of influenza vaccine (Finland only), receipt of vaccine not offered through the national immunization program to girls aged 11–14 years (Denmark, Finland, Norway only), presence of chronic disease, and season.

[c] Girls were considered unvaccinated from start of follow-up until receipt of the first dose of HPV vaccine.

[d] Girls were considered vaccinated from receipt of the first dose of HPV vaccine until the end of follow-up.

Supplementary table 10: Hazard ratios of infectious disease hospitalization according to HPV vaccination status and time since vaccination for girls in Denmark and Norway who were offered Gardasil

	Numer of cases	Person years	Incidence rate per 10 000 person years (95% CI)	Unadjusted HR (95% CI)[a]	Adjusted HR (95% CI)[b]
**DENMARK, n = 253 450[c]**					
Unvaccinated[d]	1842	365 025	50.5 (48.2, 52.8)	1 (Ref)	1 (Ref)
Vaccinated[e]	2308	554 228	41.6 (40.0, 43.4)	0.59 (0.53, 0.67)	0.82 (0.73, 0.92)
**NORWAY, n = 173 473[c]**					
Unvaccinated[d]	1219	276 432	44.1 (41.7, 46.6)	1 (Ref)	1 (Ref)
Vaccinated[e]	1348	399 336	33.8 (32.0, 35.6)	0.62 (0.52, 0.73)	0.78 (0.67, 0.90)

HPV: human papillomavirus; CI: confidence interval; HR: hazard ratio.

[a] Stratified Cox model with birth year as strata and age in days as the underlying time scale.

[b] Adjusted for household income, number of children in the household, having a single parent, maternal educational level, immigrant background, previous infectious disease hospitalization, number of previous antibiotic treatment episodes, receipt of diphtheria, tetanus, acellular pertussis booster (Finland, Sweden only), receipt of diphtheria, tetanus, acellular pertussis, inactivated polio booster (Norway only), receipt of measles, mumps, rubella vaccine (Denmark, Norway only), receipt of influenza vaccine (Finland only), receipt of vaccine not offered through the national immunization program to girls aged 11–14 years (Denmark, Finland, Norway only), presence of chronic disease, and season.

[c] Analyses restricted to birth cohorts offered Gardasil® (1996–2003 in Denmark, 1999–2004 in Norway). Girls were censored at receipt of the first dose of HPV vaccine if this occurred after the vaccine used in the program changed to Cervarix® (February 1, 2016, in Denmark; start of school year 2017/2018 in Norway).

[d] Girls were considered unvaccinated from start of follow-up until receipt of the first dose of HPV vaccine.

[e] Girls were considered vaccinated from receipt of the first dose of HPV vaccine until the end of follow-up.

Supplementary table 11: Hazard ratios of infectious disease hospitalization according to number of HPV vaccine doses and time since vaccination for girls in Denmark, Finland, Norway, and Sweden

	Numer of cases	Person years	Incidence rate per 10 000 person years (95% CI)	Unadjusted HR (95% CI)[a]	Adjusted HR (95% CI)[b]
**DENMARK, n = 253 450[c]**					
**Unvaccinated[d]**	1842	365 025	50.5 (48.2, 52.8)	1 (Ref)	1 (Ref)
**1 dose[e]**					
1–14 days since vaccination	25	8111	30.8 (20.8, 45.6)	0.56 (0.37, 0.84)	0.67 (0.45, 1.01)
15–90 days since vaccination	116	33 680	34.4 (28.7, 41.3)	0.63 (0.51, 0.77)	0.77 (0.62, 0.95)
91–180 days since vaccination	52	13 687	38.0 (29.0, 49.9)	0.74 (0.55, 0.99)	0.91 (0.68, 1.21)
181–365 days since vaccination	64	12 203	52.4 (41.0, 67.0)	0.90 (0.68, 1.19)	1.09 (0.83, 1.43)
> 365 days since vaccination	116	21 250	54.6 (45.5, 65.5)	0.79 (0.63, 0.99)	0.99 (0.79, 1.24)
**2 doses[f]**					
1–14 days since vaccination	29	7602	38.1 (26.5, 54.9)	0.66 (0.46, 0.97)	0.85 (0.58, 1.24)
15–90 days since vaccination	147	40 788	36.0 (30.7, 42.4)	0.61 (0.50, 0.74)	0.79 (0.64, 0.96)
91–180 days since vaccination	116	29 278	39.6 (33.0, 47.5)	0.64 (0.52, 0.80)	0.84 (0.68, 1.05)
181–365 days since vaccination	106	25 813	41.1 (33.9, 49.7)	0.67 (0.53, 0.83)	0.89 (0.71, 1.12)
> 365 days since vaccination	215	49 221	43.7 (38.2, 49.9)	0.60 (0.49, 0.73)	0.84 (0.69, 1.01)
**3 doses[g]**					
1–14 days since vaccination	16	5836	27.4 (16.8, 44.8)	0.42 (0.25, 0.69)	0.56 (0.34, 0.93)
15–90 days since vaccination	109	31 505	34.6 (28.7, 41.7)	0.50 (0.40, 0.63)	0.68 (0.54, 0.86)
91–180 days since vaccination	149	36 874	40.4 (34.4, 47.4)	0.56 (0.46, 0.69)	0.79 (0.65, 0.98)
181–365 days since vaccination	300	74 030	40.5 (36.2, 45.4)	0.53 (0.44, 0.63)	0.78 (0.65, 0.92)

> 365 days since vaccination	748	164 349	45.5 (42.4, 48.9)	0.52 (0.44, 0.61)	0.79 (0.67, 0.94)
**FINLAND, n = 138 261[c]**					
**Unvaccinated[d]**	698	166 824	41.8 (38.8, 45.1)	1 (Ref)	1 (Ref)
**1 dose[e]**					
1–14 days since vaccination	5	3430	14.6 (6.1, 35.0)	0.35 (0.14, 0.85)	0.36 (0.15, 0.88)
15–90 days since vaccination	13	5566	23.4 (13.6, 40.2)	0.55 (0.32, 0.96)	0.58 (0.33, 1.00)
> 90 days since vaccination[h]	8	2543	31.5 (15.7, 62.9)	0.67 (0.33, 1.35)	0.74 (0.36, 1.49)
**2 doses[f]**					
1–14 days since vaccination	10	3273	30.6 (16.4, 56.8)	0.72 (0.38, 1.36)	0.76 (0.40, 1.42)
15–90 days since vaccination	37	17 240	21.5 (15.6, 29.6)	0.49 (0.35, 0.70)	0.55 (0.39, 0.78)
91–180 days since vaccination	52	16 002	32.5 (24.8, 42.6)	0.73 (0.54, 0.99)	0.82 (0.61, 1.11)
181–365 days since vaccination	15	3812	39.3 (23.7, 65.3)	0.87 (0.51, 1.46)	0.92 (0.54, 1.55)
> 365 days since vaccination	20	3957	50.5 (32.6, 78.4)	0.90 (0.55, 1.47)	0.99 (0.60, 1.61)
**3 doses[g]**					
0–90 days since vaccination[i]	38	16 984	22.4 (16.3, 30.7)	0.50 (0.35, 0.71)	0.56 (0.39, 0.79)
91–180 days since vaccination	41	15 810	25.9 (19.1, 35.2)	0.57 (0.40, 0.80)	0.63 (0.45, 0.88)
181–365 days since vaccination	91	28 728	31.7 (25.8, 38.9)	0.66 (0.50, 0.86)	0.73 (0.56, 0.95)
> 365 days since vaccination	157	39 259	40.0 (34.2, 46.8)	0.67 (0.52, 0.86)	0.74 (0.57, 0.96)
**NORWAY, n = 173 473[c]**					
**Unvaccinated[d]**	1219	276 432	44.1 (41.7, 46.6)	1 (Ref)	1 (Ref)
**1 dose[e]**					
1–14 days since vaccination	14	5793	24.2 (14.3, 40.8)	0.56 (0.33, 0.96)	0.62 (0.36, 1.05)
15–90 days since vaccination	91	23 501	38.7 (31.5, 47.6)	0.88 (0.69, 1.13)	0.96 (0.75, 1.23)
91–180 days since vaccination	13	1382	94.1 (54.6, 162.0)	1.96 (1.00, 3.83)	1.64 (0.84, 3.22)
181–365 days since vaccination	8	872	91.8 (45.9, 183.5)	1.76 (0.65, 4.71)	1.47 (0.53, 4.08)
> 365 days since vaccination	12	1916	62.6 (35.6, 110.3)	1.03 (0.49, 2.17)	0.85 (0.37, 1.93)

**2 doses[f]**					
1–14 days since vaccination	14	5742	24.4 (14.4, 41.2)	0.54 (0.32, 0.93)	0.56 (0.33, 0.97)
15–90 days since vaccination	97	31 124	31.2 (25.5, 38.0)	0.66 (0.52, 0.85)	0.69 (0.54, 0.89)
91–180 days since vaccination	60	22 189	27.0 (21.0, 34.8)	0.54 (0.40, 0.73)	0.62 (0.46, 0.83)
181–365 days since vaccination	14	2340	59.8 (35.4, 101.0)	1.09 (0.63, 1.88)	1.05 (0.62, 1.80)
> 365 days since vaccination	18	3384	53.2 (33.5, 84.4)	0.86 (0.51, 1.45)	0.89 (0.53, 1.49)
**3 doses[g]**					
1–14 days since vaccination	15	5630	26.6 (16.1, 44.2)	0.52 (0.31, 0.89)	0.65 (0.38, 1.10)
15–90 days since vaccination	67	30 543	21.9 (17.3, 27.9)	0.42 (0.31, 0.57)	0.59 (0.44, 0.80)
91–180 days since vaccination	92	36 118	25.5 (20.8, 31.2)	0.46 (0.35, 0.61)	0.65 (0.50, 0.85)
181–365 days since vaccination	259	74 063	35.0 (31.0, 39.5)	0.61 (0.49, 0.76)	0.76 (0.61, 0.95)
> 365 days since vaccination	574	154 738	37.1 (34.2, 40.3)	0.59 (0.48, 0.72)	0.86 (0.70, 1.06)
**SWEDEN, n = 101 153[j]**					
**Unvaccinated[d]**	724	206 129	35.1 (32.7, 37.8)	1 (Ref)	1 (Ref)
**1 dose[e]**					
1–14 days since vaccination	5	3122	16.0 (6.7, 38.5)	0.47 (0.19, 1.14)	0.51 (0.21, 1.23)
15–90 days since vaccination	43	16 756	25.7 (19.0, 34.6)	0.75 (0.54, 1.03)	0.78 (0.56, 1.08)
91–180 days since vaccination	46	19 380	23.7 (17.8, 31.7)	0.65 (0.47, 0.90)	0.68 (0.49, 0.94)
181–365 days since vaccination	20	6753	29.6 (19.1, 45.9)	0.75 (0.44, 1.28)	0.78 (0.46, 1.35)
> 365 days since vaccination	15	6335	23.7 (14.3, 39.3)	0.51 (0.26, 0.99)	0.56 (0.29, 1.11)
**2 doses[f]**					
1–14 days since vaccination	5	2904	17.2 (7.2, 41.4)	0.44 (0.18, 1.08)	0.49 (0.20, 1.20)
15–90 days since vaccination	26	15 700	16.6 (11.3, 24.3)	0.42 (0.28, 0.64)	0.51 (0.33, 0.77)
91–180 days since vaccination	36	18 362	19.6 (14.1, 27.2)	0.47 (0.33, 0.68)	0.57 (0.39, 0.82)
181–365 days since vaccination	74	34 060	21.7 (17.3, 27.3)	0.49 (0.36, 0.66)	0.54 (0.40, 0.73)

> 365 days since vaccination	142	69 394	20.5 (17.4, 24.1)	0.43 (0.32, 0.57)	0.53 (0.40, 0.70)

HPV: human papillomavirus; CI: confidence interval; HR: hazard ratio

[a] Stratified Cox model with birth year as strata and age in days as the underlying time scale.

[b] Adjusted for household income, number of children in the household, having a single parent, maternal educational level, immigrant background, previous infectious disease hospitalization, number of previous antibiotic treatment episodes, receipt of diphtheria, tetanus, acellular pertussis booster (Finland, Sweden only), receipt of diphtheria, tetanus, acellular pertussis, inactivated polio booster (Norway only), receipt of measles, mumps, rubella vaccine (Denmark, Norway only), receipt of influenza vaccine (Finland only), receipt of vaccine not offered through the national immunization program to girls aged 11–14 years (Denmark, Finland, Norway only), presence of chronic disease, and season.

[c] Analyses restricted to birth cohorts eligible for three-dose schedule (1996–2003 in Denmark, 1998–2003 in Finland, 1999–2004 in Norway). Girls were censored at receipt of the first dose of HPV vaccine if this occurred after the two-dose schedule was implemented (February 1, 2016, in Denmark; start of school year 2016/2017 in Finland; start of school year 2017/2018 in Norway).

[d] Girls were considered unvaccinated (0 doses) until receipt of the first dose of HPV vaccine.

[e] Girls contributed person time in the 1-dose categories from receipt of dose 1 until receipt of dose 2 or end of follow-up.

[f] Girls contributed person time in the 2-dose categories from receipt of dose 2 until receipt of dose 3 or end of follow-up.

[g] Girls contributed person time in the 3-dose categories from receipt of dose 3 until end of follow-up.

[h] The categories 91–180 days since dose 1, 181–365 days since dose 1, and > 365 days since dose 1 were combined due to few cases.

[i] The categories 1–14 days since dose 3 and 15–90 days since dose 3 were combined due to few cases.

[j] Girls were censored at receipt of the third dose, since the birth cohorts included in the study population were eligible for two-dose schedule.

Supplementary table 12: Hazard ratios of infectious disease hospitalization comparing girls who received MMR vaccine together with the first dose of HPV vaccine to girls who received MMR vaccine before the first dose of HPV vaccine

	Numer of cases	Person years	Incidence rate per 10 000 person years (95% CI)	Unadjusted HR (95% CI)[a]	Adjusted HR (95% CI)[b]
**DENMARK, n = 199 265**					
**HPV1 after MMR**[c]	586	121 178	48.4 (44.6, 52.4)	1 (Ref)	1 (Ref)
**HPV1 + MMR**[d]	1463	378 081	38.7 (36.8, 40.7)	0.96 (0.86, 1.08)	1.01 (0.90, 1.14)

MMR: measles, mumps; HPV: human papillomavirus; CI: confidence interval; HR: hazard ratio.

[a] Stratified Cox model with birth year as strata and age in days as the underlying time scale.

[b] Adjusted for household income, number of children in the household, having a single parent, maternal educational level, immigrant background, previous infectious disease hospitalization, number of previous antibiotic treatment episodes, receipt of vaccine not offered through the national immunization program to girls aged 11–14 years, presence of chronic disease, season, and receipt of additional doses of HPV vaccine.

[c] First dose of HPV vaccine received after MMR vaccine.

[d] First dose of HPV vaccine received together with MMR vaccine.

References

1. Lubker CL, Lynge E. Stronger responders-uptake and decline of HPV-vaccination in Denmark. Eur J Public Health. 2019;29:500-5.

2. Statens Serum Institut. EPI-NYT. No 35. 2008. https://en.ssi.dk/-/media/arkiv/uk/news/epi-news/2008/pdf/epi-news---2008---no-35.pdf?la=en.

3. Statens Serum Institut. EPI-NYT. No 26-33. 2003. https://en.ssi.dk/-/media/arkiv/uk/news/epi-news/2003/pdf/epi-news---2003---no-26-33.pdf?la=en.

4. Statens Serum Institut. EPI-NYT. No 23. 2001. https://en.ssi.dk/-/media/arkiv/uk/news/epi-news/2001/pdf/epi-news---2001---no-23.pdf?la=en.

5. Statens Serum Institut. EPI-NYT. No 24. 2003. https://en.ssi.dk/-/media/arkiv/uk/news/epi-news/2003/pdf/epi-news---2003---no-24.pdf?la=en.

6. Statens Serum Institut. EPI-NYT. No. 9. 2008. https://en.ssi.dk/-/media/arkiv/uk/news/epi-news/2008/pdf/epi-news---2008---no-9.pdf?la=en.

7. Finnish Institute for health and welfare. När inleddes de olika vaccinationerna i Finland? (In Swedish). https://thl.fi/sv/web/infektionssjukdomar-och-vaccinationer/information-om-vaccinationer/det-nationella-vaccinationsprogrammet/nar-inleddes-de-olika-vaccinationerna-i-finland-. (Accessed June 21, 2024).

8. Ministry of Social Affairs and Health. HPV vaccinations. https://stm.fi/en/hpv-vaccinations. (Accessed June 21, 2024).

9. Finnish Institute for health and welfare. Diphtheria, tetanus and whooping cough vaccine (DTaP) for adolescents and adults. In Swedish. https://thl.fi/en/web/infectious-diseases-and-vaccinations/vaccines-a-to-z/diphtheria-tetanus-whooping-cough-polio-and-hib-combination-vaccines/diphtheria-tetanus-and-whooping-cough-vaccine-dtap-for-adolescents-and-adults. (Accessed June 21, 2024).

10. Norwegian Institute of Public Health. Barnevaksinasjonsprogrammet i Norge. Rapport for perioden 2001-2010. 2012. https://www.fhi.no/globalassets/dokumenterfiler/rapporter/vaksine/barnevaksinasjonsprogrammet.-rapport-for-perioden-2001-2010.pdf. (In Norwegian).

11. The Norwegian Institute of Public Health. Barnevaksinasjonsprogrammet i Norge. Rapport for 2013. 2014. https://www.fhi.no/globalassets/dokumenterfiler/rapporter/vaksine/barnevaksinasjonsprogrammet-i-norge-rapport-2013. (In Norwegian).

12. Public Health Agency of Sweden. Vaccinationsprogram för barn. En kunskapsöversikt för hälsovårdspersonal. 2018. https://www.folkhalsomyndigheten.se/publicerat-material/publikationsarkiv/v/vaccination-av-barn-det-svenska-vaccinationsprogrammet-en-kunskapsoversikt-for-halsovardspersonal/. (In Swedish).

13. Wang J, Ploner A, Sparen P, et al. Mode of HPV vaccination delivery and equity in vaccine uptake: A nationwide cohort study. Prev Med. 2019;120:26-33.

14. Gehrt L, Laake I, Englund H, et al. Hospital Contacts for Infectious Diseases Among Children in Denmark, Finland, Norway, and Sweden, 2008-2017. Clin Epidemiol. 2022;14:609-21.

15. Skajaa N, Gehrt L, Nieminen H, et al. Trends in Antibiotic Use in Danish, Finnish, Norwegian and Swedish Children. Clin Epidemiol. 2022;14:937-47.

16. Kristensen K, Hjuler T, Ravn H, Simoes EA, Stensballe LG. Chronic diseases, chromosomal abnormalities, and congenital malformations as risk factors for respiratory syncytial virus hospitalization: a population-based cohort study. Clin Infect Dis. 2012;54:810-7.
